# Supplementary material for: Divergent effects of healthy ageing on semantic knowledge and control: Evidence from novel comparisons with semantically impaired patients
Source: J Neuropsychol. 2018 Apr 17;13(3):462–84. doi: 10.1111/jnp.12159 (PMC6766984; doi:10.1111/jnp.12159)

*Supplementary Figure 1: Correlations between accuracy on trials of the task and the psycholinguistic predictors and patient performance indices*


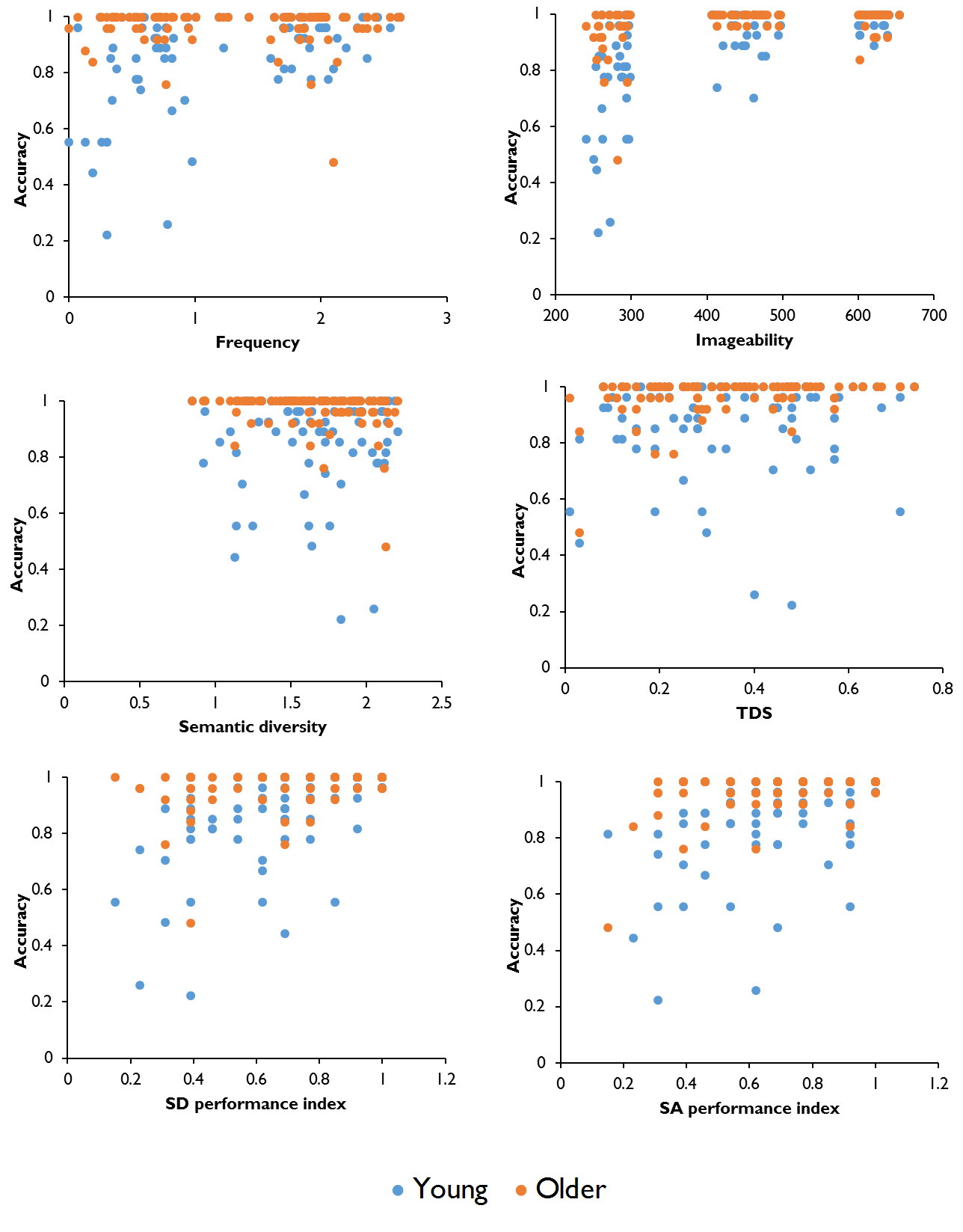

Supplement: Supplementary file 1 — Figure S1. Correlations between accuracy on trials of the task and the psycholinguistic predictors and patient performance indices. [file JNP-13-462-s001.docx]
